# Retraction Note: MicroRNA-130b promotes lung cancer progression via PPARγ/VEGF-A/BCL-2-mediated suppression of apoptosis

**DOI:** 10.1186/s13046-022-02548-2

**Published:** 2022-12-17

**Authors:** Jianwei Tian, Liping Hu, Xiao Li, Jian Geng, Meng Dai, Xiaoyan Bai

**Affiliations:** 1 State Key Laboratory for Organ Failure Research, Division of Nephrology, Nanfang Hospital, Southern Medical University, Guangzhou, 510515 Guangdong China; 2 Department of Emergency, Nanfang Hospital, Southern Medical University, Guangzhou, Guangdong China; 3 Department of Pathology, Nanfang Hospital, Southern Medical University, Guangzhou, Guangdong China; 4 Health Management Center, Nanfang Hospital, Southern Medical University, Guangzhou, Guangdong China

**Retraction Note:** ***J Exp Clin Cancer Res*** **35, 105 (2016)**

**https://doi.org/10.1186/s13046-016-0382-3**

The authors have retracted this article due to irregularities found in figures 3G and J, 4G and J, 5G and J and 6F. The authors therefore no longer have confidence in the reliability of their results. Xiaoyan Bai has not explicitly stated whether they agree to this retraction notice. Jianwei Tian, Liping Hu, Xiao Li, Jian Geng and Meng Dai have not responded to any correspondence from the editor/publisher about this retraction.

